# Supplementary material for: Cellular hallmarks reveal restricted aerobic metabolism at thermal limits
Source: eLife. 2015 May 1;4:e04810. doi: 10.7554/eLife.04810 (PMC4415524; doi:10.7554/eLife.04810)
Supplement: Figure 1—source data 1. — List of features that were quantified and their thermal responses within and beyond the thermal range for C. elegans (N2). Within the thermal range, features were categorized as ‘temperature-dependent’ if the Pearson correlation p-value was below 0.0014 = 0.05/35 (see ‘Materials and methods’ for Bonferroni correction; ‘temperature-independent’ is shown underlined). Beyond the thermal limit, we performed an F-test to determine if the thermal response of the feature was changing compared to within the thermal range (see ‘Materials and methods’; we indicated a change in thermal response when the F-test p-value was below 0.0014, highlighted in bold). Abbreviations: PC: pseudo-cleavage, PM: pronuclear meeting, ME: mitotic entry, T: temperature, C/R: centration-rotation, MT: microtubules. The following features were also quantified but displayed no consistent thermal response both within and beyond the thermal range and hence were not included in the table: anterior-most position at the end of C/R, number of anterior and posterior oscillations, spindle position at the onset of oscillations. DOI: http://dx.doi.org/10.7554/eLife.04810.004 [file elife04810s001.docx]

**Figure 1 - Source data 1.**  Quantified features.

| \| *Feature* \| *Below lower thermal limit* \| *Within thermal range* \| *Above upper thermal limit* \| \| --- \| --- \| --- \| --- \| \| CELL CYCLE DURATION \|  \|  \|  \| \| Timing PC \| **T-independent** \| T-dependent \| *unchanged* \| \| Timing PM \| **T-independent** \| T-dependent \| *unchanged* \| \| Timing ME \| **T-independent** \| T-dependent \| **T-independent** \| \| Relative timing PC-PM \| *unchanged* \| T-independent \| *unchanged* \| \| Relative timing PM-ME \| *unchanged* \| T-independent \| *unchanged* \| \| Relative timing ME-CK \| *unchanged* \| T-independent \| *unchanged* \| \| PRONUCLEI MIGRATION \|  \|  \|  \| \| Female pronucleus speed \| *unchanged* \| T-dependent \| **(significantly more) T-dependent** \| \| Female pronucleus acceleration \| *unchanged* \| T-dependent \| **(significantly more) T-dependent** \| \| Timing max speed female pronucleus \| **T-independent** \| T-dependent \| *unchanged* \| \| Distance traveled from PC to PM \| **T-independent** \| Inversely T-dependent \| *unchanged* \| \| CENTRATION/  ROTATION \|  \|  \|  \| \| Max speed centration \| *unchanged* \| T-dependent \| *unchanged* \| \| Max angular speed rotation \| *unchanged* \| T-dependent \| *unchanged* \| \| Time from the end of C/R to the start of spindle pole oscillations \| **T-independent** \| Inversely T-dependent \| *unchanged* \| \| Timing of max speed centration \| **T-independent** \| T-dependent \| *unchanged* \| \| Timing of max speed rotation \| **T-independent** \| T-dependent \| *unchanged* \| \| SPINDLE ELONGATION \|  \|  \|  \| \| Relative spindle mid-position (% embryo length) \| *unchanged* \| T-dependent \| **(significantly more) T-dependent** \| \| Max aster elongation speed towards anterior pole \| *unchanged* \| T-dependent \| *unchanged* \| \| Max aster elongation speed towards posterior pole \| *unchanged* \| T-dependent \| *unchanged* \| \| SPLINDLE ROCKING \|  \|  \|  \| \| Posterior pole oscillations frequency \| **T-independent** \| T-dependent \| *unchanged* \| \| Max amplitude of posterior oscillations \| *unchanged* \| T-independent \| **T-independent (decrease at 27°C)** \| \| Duration of posterior oscillations \| **T-independent** \| Inversely T-dependent \| *unchanged* \| \| Timing of the max amplitude oscillation in posterior \| *unchanged* \| T-independent \| *unchanged* \| \| Timing of the start of posterior oscillations \| **T-independent** \| T-dependent \| **T-independent** \| \| Anterior pole oscillations frequency \| **T-independent** \| T-dependent \| *unchanged* \| \| Max amplitude of anterior oscillations \| *unchanged* \| T-independent \| *unchanged* \| \| Duration of anterior oscillations \| **T-independent** \| T-dependent \| **T-independent (increase at 27°C)** \| \| Timing of the max amplitude oscillation in anterior \| *unchanged* \| T-independent \| *unchanged* \| \| Timing of the start of anterior oscillations \| *unchanged* \| T-dependent \| *unchanged* \| \| SIZE AND ASYMMETRY \|  \|  \|  \| \| Relative area of AB \| **T-dependent** \| T-independent \| **T-dependent** \| \| Relative area of P1 \| **T-dependent** \| T-independent \| **T-dependent** \| \| Embryo size \| **T-dependent** \| Inversely T-dependent \| **T-independent** \| \| Egg size \| **T-dependent** \| Inversely T-dependent \| **T-independent** \| \| Major axis length \| **T-dependent** \| Inversely T-dependent \| **T-dependent** \| \| Embryo elongation (major/minor axes) \| *unchanged* \| Inversely T-dependent \| **T-dependent** \| |
| --- | --- | --- | --- | --- | --- | --- | --- | --- | --- | --- | --- | --- | --- | --- | --- | --- | --- | --- | --- | --- | --- | --- | --- | --- | --- | --- | --- | --- | --- | --- | --- | --- | --- | --- | --- | --- | --- | --- | --- | --- | --- | --- | --- | --- | --- | --- | --- | --- | --- | --- | --- | --- | --- | --- | --- | --- | --- | --- | --- | --- | --- | --- | --- | --- | --- | --- | --- | --- | --- | --- | --- | --- | --- | --- | --- | --- | --- | --- | --- | --- | --- | --- | --- | --- | --- | --- | --- | --- | --- | --- | --- | --- | --- | --- | --- | --- | --- | --- | --- | --- | --- | --- | --- | --- | --- | --- | --- | --- | --- | --- | --- | --- | --- | --- | --- | --- | --- | --- | --- | --- | --- | --- | --- | --- | --- | --- | --- | --- | --- | --- | --- | --- | --- | --- | --- | --- | --- | --- | --- | --- | --- | --- | --- | --- | --- | --- | --- | --- | --- | --- | --- | --- | --- | --- | --- | --- | --- | --- | --- | --- | --- | --- | --- | --- |

**Figure 1 - Source data 1.** Quantified features. List of features that were quantified and their thermal responses within and beyond the thermal range for *C. elegans* (N2). Within the thermal range, features were categorized as “temperature-dependent” if the Pearson correlation p-value was below 0.0014=0.05/35 (see Supplementary File 1 for Bonferroni correction; “temperature-independent” is shown underlined). Beyond the thermal limit, we performed an F-test to determine if the thermal response of the feature was changing compared to within the thermal range (see Supplementary File 1; we indicated a change in thermal response when the F-test p-value was below 0.0014, **highlighted in bold**). Abbreviations: PC: pseudo-cleavage, PM: pronuclear meeting, ME: mitotic entry, T: temperature, C/R: centration-rotation, MT: microtubules. The following features were also quantified but displayed no consistent thermal response both within and beyond the thermal range and hence were not included in the table: anterior-most position at the end of C/R, number of anterior and posterior oscillations, spindle position at the onset of oscillations.
